# Nucleic Acid Nanotechnology for Diagnostics and Therapeutics in Acute Kidney Injury

**DOI:** 10.3390/ijms23063093

**Published:** 2022-03-13

**Authors:** Yiwen Ying, Qian Tang, Da Han, Shan Mou

**Affiliations:** 1 Department of Nephrology, Renji Hospital, School of Medicine, Shanghai Jiao Tong University, Shanghai 200127, China; yingyiwen0813@163.com; 2 Institute of Molecular Medicine, Shanghai Key Laboratory for Nucleic Acid Chemistry and Nanomedicine, Renji Hospital, School of Medicine, Shanghai Jiao Tong University, Shanghai 200127, China; qtang@sjtu.edu.cn

**Keywords:** nucleic acid nanotechnology, AKI, aptamer, framework nucleic acids, diagnosis, targeted therapy, biomarkers

## Abstract

Acute kidney injury (AKI) has impacted a heavy burden on global healthcare system with a high morbidity and mortality in both hospitalized and critically ill patients. However, there are still some shortcomings in clinical approaches for the disease to date, appealing for an earlier recognition and specific intervention to improve long-term outcomes. In the past decades, owing to the predictable base-pairing rule and highly modifiable characteristics, nucleic acids have already become significant biomaterials for nanostructure and nanodevice fabrication, which is known as nucleic acid nanotechnology. In particular, its excellent programmability and biocompatibility have further promoted its intersection with medical challenges. Lately, there have been an influx of research connecting nucleic acid nanotechnology with the clinical needs for renal diseases, especially AKI. In this review, we begin with the diagnostics of AKI based on nucleic acid nanotechnology with a highlight on aptamer- and probe-functionalized detection. Then, recently developed nanoscale nucleic acid therapeutics towards AKI will be fully elucidated. Furthermore, the strengths and limitations will be summarized, envisioning a wiser and wider application of nucleic acid nanotechnology in the future of AKI.

## 1. Introduction

Acute kidney injury (AKI) is a clinical syndrome characterized by an abrupted decline in renal function due to miscellaneous factors, such as rapid volume depletion, acute infection, nephrotoxic medicines and so on, leading to a retention of nitrogen wastes and creatinine accompanied by electrolyte disturbances and acid-base imbalance [1,2]. Accordingly, AKI occurs in more than 50% of patients in intensive care unit (ICU) while mortality reaches 10~20% even in non-ICU hospitalized patients suffering AKI, spawning huge significance to its early identification and medical intervention [3,4]. However, current clinical approaches are complicated with several drawbacks. For instance, serum creatinine, the mostly used index to evaluate renal status, is easily affected by individual differences, hindering its accuracy as a diagnostic method [5]. Other commonly utilized detection methods may also require much time as well as skillful techniques [6]. As for AKI therapies, mainly symptomatic treatment is provided without consistent standards or specific medicines [7]. Hence, given the fast progression and life threatening feature of AKI, it calls for more rapid and efficient diagnostic and therapeutic strategies [4].

**Figure 1:**
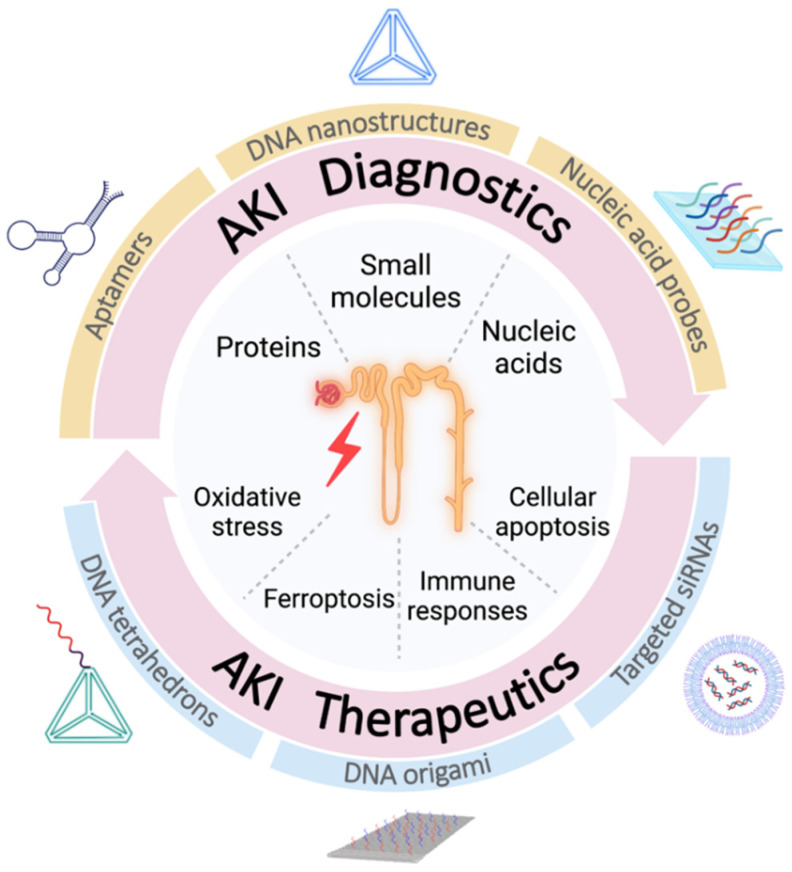
Schematic diagram of how nucleic acid nanotechnology plays a part in the diagnostic and therapeutic strategies of acute kidney injury (AKI). In AKI diagnostics, aptamers, DNA nanostructures as well as nucleic acid probes can be functionalized to detect AKI-related protein biomarkers, small molecules and nucleic acids. Meanwhile, in AKI therapeutics, oxidative stress, ferroptosis, immune responses and cellular apoptosis can be targeted with DNA tetrahedrons, DNA origamis and well-directed short interference RNAs (siRNAs). Created with BioRender.com (26 February 2022).

Nucleic acids have no longer been limited to the definition of genetic information carrier. Originated from Dr. Seeman’s idea in the 1980s, nucleic acids have turned out to be unique but reliable bricks for biological nanomaterials realized by the predictable Watson-Crick base pairing rule and infinite combinations and permutations [8,9]. In the past decade, the concept of nucleic acid nanotechnology has further blossomed in the field of biomedical science, including smart drug delivery systems and molecular recognition tools [10,11]. Its advantages in disease diagnosis can be attributed to the precise recognition, tailored modifications and abundant amplification strategies while nucleic acid nanotechnology-based therapeutics benefit from advantages of structural programmability, spatial addressability and excellent biocompatibility [12,13]. Recently, an increasing number of research focus on the applications of nucleic acid nanotechnology in nephrological diseases as the inborn renal clearance endows kidneys with prominent biodistribution of certain DNA nanostructures over other organs, setting foundation for the kidney-targeting methods with nucleic acid nanotechnology [14]. Herein, nucleic acid nanotechnology-based diagnostics and therapeutics in AKI are summarized and elaborated, respectively, each with several subsections introducing different AKI diagnostic targets as well as AKI nanomedicines based on nucleic acids (Figure 1).

## 2. Diagnostics of AKI Based on Nucleic Acid Nanotechnology

Despite the fact that nucleic acid nanotechnology has been extensively investigated in cancer diagnosis, its research in AKI detection is still on the rise, among which aptamer may emerge as a robust tool for the disease diagnosis [10]. Aptamer, also known as chemical antibody, is a single stranded DNA or RNA (ssDNA/ssRNA) molecule which possesses high specificity as well as affinity towards multifarious ligands, from macromolecules such as proteins, to micro molecules such as peptides, amino acids, and metal ions [15,16,17]. These outstanding characteristics form the prerequisite for novel biosensing platforms while integration with new biomaterials magnifies the advantages of aptamers, boosting both sensitivity and specificity of in-time disease diagnosis, especially for AKI which urges earlier judgment and intervention [18]. In this section, we mainly introduce nucleic acid nanotechnology-based diagnostics targeting AKI-related proteins, small molecules and nucleic acids, particularly on their detection performance and how aptamers or nucleic acid probes are incorporated into various methodologies thus facilitating AKI diagnosis. In addition, non-aptamer/probe aided diagnostic methods indirectly implying AKI and modified DNA-mediated AKI proteomic discoveries are also discussed in the following paragraphs (Table 1).

### 2.1. AKI Diagnostics Targeting Proteins

#### 2.1.1. NGAL

Neutrophil gelatinase-associated lipocalin (NGAL), also called lipocalin 2 (LCN2), is a 25-kDa protein which is used as a marker of acute kidney damage and mainly released by renal tubular cells during an insult [36]. It is reported that urinary and serum NGAL protein as well as its mRNA level elevated significantly in the early phase of various kinds of AKI, much faster than serum creatinine [37,38,39,40]. It is also suggested that NGAL upregulation may conduct renoprotective effects [41]. A sensitive and specific NGAL detection can bring sufficient therapeutic window for patients suffering AKI.

Aptamers can be utilized to detect NGAL in AKI together with rival antibodies. In 2019, Hong et al. [19] revealed an aptamer via SELEX, named NA53, as the most optimal chemical antibody against NGAL. Herein, SELEX refers to systematic evolution of ligands by exponential enrichment, which is a standard method for selecting oligonucleotide sequences of ssDNA/RNA that can specifically bind to a certain target ligand in vitro from a random nucleotide library [42]. Hong et al. established an enzyme-linked aptamer analysis (ELAA) method, in which NGAL antibodies were coated in a 96-well plate, together with a subsequently added NGAL protein and biotin-modified NA53 aptamer forming a sandwich-like structure. The NA53-based ELAA method proved its ability of accurately distinguishing AKI patients from healthy people with a sensitivity of 100% and specificity of 90% in clinical urine samples without unspecific interactions with globulins or albumins. The limit of detection (LOD) was 30.45 ng/mL, which was comparable to the current standard method for detecting NGAL.

**Figure 2:**
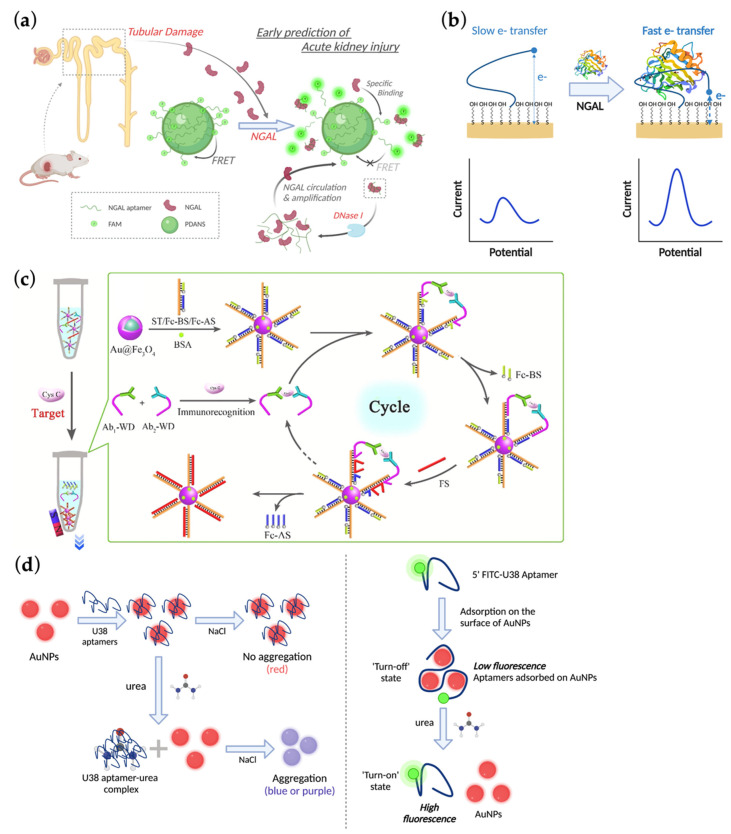
Mechanisms involved in aptamer-based detection of acute kidney injury (AKI)-related proteins and small molecules. (**a**) Schematic illustration of how specific binding between polydopamine nanosphere-attached aptamers and AKI biomarker neutrophil gelatinase-associated lipocalin (NGAL) turns on the fluorescence signal and amplified with the help of DNase I-mediated NGAL circulation. Adapted with permission from [20] and created with BioRender.com (27 February 2022). Copyright © 2022, Royal Society of Chemistry; (**b**) Diagrammatic plot of the relationship between the current signal within an electrochemical aptamer-based sensor and the DNA strand folding induced by specific NGAL binding. Adapted with permission from [22] and created with BioRender.com (27 February 2022). Copyright © 2022, American Chemical Society; (**c**) Detailed mechanism of how antibody-cystatin C recognition induces a repeating series of DNA strand replacement through a 3D DNA machine, as well as a magnetic separation and signal detection via designed eletrochemiluminescent nanomaterials. Adapted with permission from [25]. Copyright © 2022, Elsevier; (**d**) Diagrams of two different kinds of gold nanoparticle-functionalized aptasensors detecting urea based on colorimetric changes (left) and fluorescence quenching/restoration (right), respectively. Adapted with permission from [31] and created with BioRender.com (27 February 2022). Copyright © 2022, Elsevier.

A polydopamine nanosphere (PDANS) carrier combined with 5-carboxyfluorescein (FAM) labelled NGAL-specific aptamer presented by Tian and Yu’s team showed another perspective of nucleic acid nanotechnology-based protein detection [20] (Figure 2a). In this model, the quenched fluorescence of FAM was recovered once the aptamer was dissociated from the surface of PDANS by binding with NGAL [43]. Of note, with the help of DNase I, the dissociated aptamer was digested to release NGAL for target recycling, thus effectively amplifying the FAM signal and remarkably improving LOD to 6.25 pg/mL. The NGAL-reporting ability of this DNase I-assisted nanoassembly was verified in both intracellular environment and urine sample of AKI mice. Moreover, the detection process required about only 1 h. Together with its simplicity and impressive sensitivity, this aptamer-based tool is promising in the early diagnosis of AKI.

Aptamer-functionalized electrochemical sensors have been increasingly developed in recent years, attributed to the characteristics that aptamers can be chemically modified in various ways to fit with advanced materials. Matassan et al. [21] introduced an amine-modified NGAL aptamer to a graphene nanoplatelet-functionalized biochip surface via covalent bonding, achieving a sub-picomolar LOD of 0.07 pg/mL. In 2020, Parolo et al. [22] developed a novel electrochemical aptamer-based (EAB) sensor which could carry out real-time and continuous monitoring of urine NGAL (Figure 2b). Concretely, the researchers truncated the parent sequence of the NGAL aptamer, which destabilized the DNA and rendered it the ability of binding-induced structure folding [44]. The aptamer was further modified with a redox-reporter and employed to sensor platform, where its binding with NGAL could lead to conformational changes and cause the redox species to tether nearer to the platform, hence a faster electro transfer within the platform detected. The whole process was autonomic and required no exogenous washing agents. Aptamers played a pivotal role in EAB sensors as their high specificity conferred the platform to a stable monitoring in a complex sample stream, such as human urine in this context. LOD of this method was 2 nmol/L in artificial urine and 3.5 nmol/L in authentic urine. More recently, another research team designed an inkjet-printed electrochemical aptasensor device based on principle of impedance, in which the NGAL-specific aptamer was composed of a thiol-modified end endowing its immobilization. Through binding with the target, the impedance of device will change, resulting in signal changes that are correlated with NGAL concentrations. They combined this design with their proposal of ‘Plug, Print & Play technology’, which referred to handy equipment and easy impedance measurement via the audio input of a smartphone, realizing low-cost, simplicity and the concept of point-of-care [23]. The LOD of this device was 3 nmol/L in artificial urine. Their idea of using smartphone as the terminal data reader could bring convenience to clinicians in real life.

Apart from ssDNA aptamers mentioned above, RNA aptamers have also been used to recognize AKI biomarker according to Nilsen-Hamilton’s research [24]. They screened out an RNA aptamer that specifically bound murine Ngal (mLcn2) via the iron-siderophore binding pocket of the target protein. They then incorporated the RNA into a differential interferometry-based microcantilever sensor which could determine the mechanical deformation induced by aptamer-ligand binding. This in-line monitoring system revealed an LOD of 4 nmol/L for mLcn2.

#### 2.1.2. Cystatin C

Cystatin C (cysC) is a 13-kDa protein which is used as an indicator of kidney function [45]. This is due to the physiological nature that cysC filters through the glomerulus freely followed by entire reabsorption and catabolization without extra secretion in renal tubules [46]. CysC is less dependent of sex, age, weight and height, which can be a substitute for serum creatinine to reflect renal impairment [47,48]. During the early phase of AKI, serum cysC can increase sharply, implicating its potential in AKI in-time diagnosis [49,50].

In 2019, Ding and colleagues managed to detect cysC with ultrahigh sensitivity by developing a 3D DNA machine induced by immunorecognition [25] (Figure 2c). Firstly, they synthesized a three-stranded nanocomplex composed of a longer substrate DNA (ST) and two shorter modified strands called ferrocene-labeled blocker/assistant strand (Fc-BS/Fc-AS) on the surface of gold-functionalized Fe_3_O_4_. Next, they designed a pair of capturers targeting cysC, namely walker DNA-labeled antibody 1 and 2 (Ab_1/2_-WD). Once the DNA-linked antibody pair specifically recognized the AKI biomarker, they formed a sandwich-like conjugate and replaced the Fc-BS strands in the ST/Fc-BS/Fc-AS complex via DNA strand displacement, which was a commonly used technique in DNA nanotechnology. As a result, the originally hidden toeholds were exposed, permitting fuel strands (FS) to bind with and subsequently displacing both Fc-AS strands and the sandwich-like conjugate, which was a potential energy supply for the cysC-antibody complex to further walk along the 3D DNA machine automatically. The entire process could undergo several cycles and eventually release a large amount of Fc-AS and Fc-BS strands, followed by Fe_3_O_4_-mediated magnetic separation and eventual detection. Accordingly, the enzyme-free biosensor could detect a linear range of cysC from 1.0 fg/mL to 10 ng/mL and an LOD of 0.38 fg/mL.

There are also a few aptamer-mediated alternative ways to detect cysC, which resemble the methods mentioned in 2.1.1. Wang et al. [26] developed a robust biosensor facilitated with fluorophore-labelled aptamer and DNase I-mediated amplification strategy, yet they chose graphene oxide as the carrier and protector of the conjugated aptamers and cysC as the target protein, realizing an LOD of 0.16 ng/mL. Kooshki et al. [6] screened out a highly specific aptamer K17 via SELEX and managed to detect human serum cysC with a competitive enzyme-linked aptamer sorbent assay, which could perform better in complex context such as plasma. The LOD was 216.077 pg/mL. Natarajan et al. [27] designed a quantitative fluorescence kit for cysC based on an aptamer-antibody pair-based lateral flow assay (LFA), in which the target protein formed a sandwich-like structure together with 5’-dye-modified aptamer and cysC-specific capturing antibodies. The LOD of this aptamer-based kit was 0.023 µg/mL, which was comparable to commercially available cysC kit.

#### 2.1.3. RBP4

Retinol binding protein 4 (RBP4) is a 21-kDa protein molecule commonly filtered through kidney glomerulus and reabsorbed by tubules where kidney injury may impair the uptake of this protein, striking an increase in its urine excretion [51]. Recently, urinary RBP4 has been emerging as a functional biomarker for reflection of proximal renal tubules and early detection of AKI under several circumstances, including ischemic, septic, and post-transplantation AKI, etc. [52]. Lee et al. [28] developed a surface plasmon resonance biosensor targeting RBP4 specifically via SELEX-screened selection of ssDNA aptamers and demonstrated a better detection performance with dose-dependent responses and higher sensitivity over ELISA kits. Their research was mainly focused on RBP4 in type 2 diabetes and verified in artificial serum samples. However, RBP4 was reported with several different isoforms in serum and urine context [52]. Therefore, more work should be carried out in detecting and validating the role of RBP4 in AKI urine sample. Nevertheless, Lee’s research shed a light on improving the recognition of this protein, probably paving a new way for AKI diagnosis.

#### 2.1.4. Albumin

Albumin, one of the most mentioned serum proteins in laboratory examination, has long been used to evaluate one’s progression in chronic kidney disease (CKD) [53]. Recent studies have shown that urinary albumin can be a potential biomarker in recognizing early AKI of several subtypes, comparable to the performance of NGAL [53,54,55]. However, the mainstream methods for urinary albumin detection is dipstick, which is only semi-quantitative, and immunoturbidimetric, which may underestimate the level of albumin [56]. With regard to this challenge, a research group utilized an 87-nucleotide aptamer based on fluorescence quenching and graphene oxide, realizing a simple, precise and highly cost-effective detection of albumin in both clinical serum and urine samples [29]. Their aptasensor obtained an LOD of 0.05 μg/mL and could detect albumin ranging from 0.1 to 14 μg/mL. More recently, Cheeveewattanagul et al. [30] obtained a wider scope of urinary albumin detection (up to 400 μg/mL) through another method, covering the target from normoalbuminuria to macroalbuminuria. Concretely, aptamer-functionalized magnetic nanoparticles were applied to capture albumin specifically, followed by magnetic separation and re-dispersal, allowing the preconcentration as well as full exposure of the bound complexes to a methylene blue redox solution. As methylene blue fell in concentration by adsorbing to albumin, a second magnetic manipulation helped separate remaining redox solution from bound particles, and a decreased reduction current in the solution could be sensed via differential pulse voltammetry, which was in proportion to an elevated albumin level in clinical urine samples. In addition, the team took urinary pH into consideration and generated consistent results at pH values from 4.55 to 8.0 with calibrated mathematic formulation, which covered the urine acidity at real clinics that always varied among individuals. Furthermore, it was also claimed that all reagents in this research were amenable to mass-production scale, boosting its cost-effectiveness. 

### 2.2. AKI Diagnostics Targeting Small Molecules

Urea, more commonly referred to as blood urea nitrogen (BUN) at clinics representing the nitrogen content in urea, is a small molecule metabolized from proteins and filtered through kidneys. It can facilitate physicians to judge one’s renal function parallel to serum creatinine. Although doubted on its non-specificity similar to creatinine, fractional excretion of urea standardized to creatinine may still help differentiate transient from persistent AKI [57]. Extant methods of urea detection are mainly derived from urease enzyme, which may be limited by accuracy and reproducibility [58,59]. In 2015, Kumar et al. [31] designed non-enzymatic urea detection methods with LOD of 20 mmol/L starting from isolating urea-specific aptamers via FluMag-SELEX, which was a modified form of SELEX characterized by fluorescence-aided DNA monitoring and magnetic separation technology [60] (Figure 2d). They then designed two aptasensors on the basis of the selected aptamer U38 as well as the aggregation property of unmodified gold nanoparticles (AuNPs) in salt-containing samples. In the first nanosensor, once urea-U38 binding triggered dissociation of aptamers from AuNPs due to high specificity and affinity, the originally scattered AuNPs separated by U38 aggregated and mediated a colorimetric change from red to purple. Meanwhile, in another aptasensor, fluorescence-labelled aptamers attached to AuNPs emitted low signal due to Fluorescence Resonance Energy Transfer, yet the fluorescence restored once urea was spiked in the sample and separated U38 from the gold nanocarriers. In 2018, Mansouri and Azadbakht fabricated an electrochemical aptasensor platform with an LOD of 370 pmol/L based on ssDNA specific recognition of urea and signal amplification realized by carbon nanotubes/amine-functionalized graphene oxide. More concretely, in the presence of urea, the DNA probe formed a complex with its target and subsequently hindered the electron transfer, hence a weaker electrochemical signal recorded [32].

### 2.3. AKI Diagnostics Targeting Nucleic Acids

Nucleotide-based probe is also a simple but robust tool in the field of nucleic acid nanotechnology other than aptamers. Watson-Crick base pairing rule enables these short probes to detect complementary sequences natively. Herein, micro ribonucleic acid (miRNA), a single-stranded small non-coding RNA molecule which can regulate various gene expressions and physiological events, is mainly introduced as the biomarker for AKI [61,62]. 

**Figure 3:**
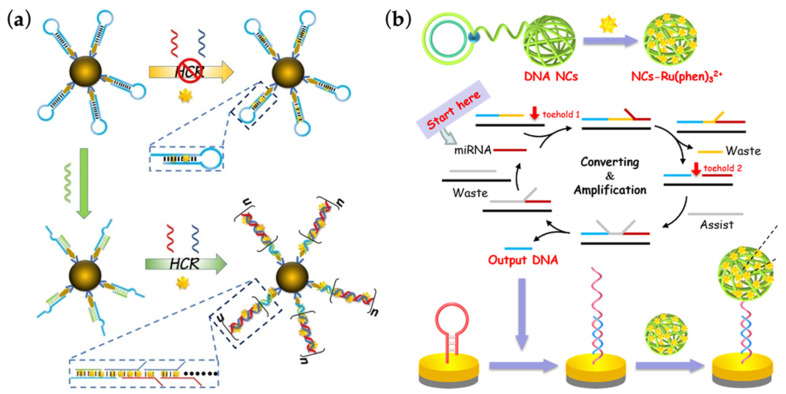
Mechanisms involved in the detection of AKI-related microRNAs (miRNA) with nucleic acid nanotechnology. (**a**) With the help of hybridization chain reaction, binding between miR-21 and capture strands leads to the exposure of a sticky end and a subsequent hybridization with other strands, eventually realizing double-stranded nucleotide elongation and an intensified loading of luminescent reporters. Adapted with permission from [33]. Copyright © 2022, Elsevier; (**b**) Schematic drawing of how toehold-mediated strand displacement facilitates the transformation and amplification of signals originating from the hybridization between miR-21 and substrate strands. Adapted with permission from [34]. Copyright © 2022, Elsevier.

It is well acknowledged that miR-21 plays a significant role in the pathogenesis of AKI [63]. Urinary level of miR-21 succeeds in distinguishing AKI patients with non-AKI ones, thus regarded as a novel biomarker in the disease [64]. Chen’s group managed to detect miR-21 in AKI urine samples via an innovative nucleic acid-based amplification method [33] (Figure 3a). Concretely, biotin-modified capture probes H1 (CPH1) which could bind miR-21 specifically were lodged on streptavidin-modified magnetic beads. In the presence of miR-21, the target could open the hairpin structure of CPH1, resulting in an exposure of the sticky end which could further hybridize with another DNA strand H2. As the extended part of H2 was designed with a specific sequence identical to CPH1, it in turn hybridized with probe H1, leading to a repeated process of H1- and H2-mediated double-stranded nucleotide elongation, which was also called a hybridization chain reaction (HCR). With luminescent indicators embedded in dsDNA grooves in the meantime, the whole amplification procedure with intensified signals could be detected. Of note, the magnetic beads mentioned here not only facilitated enrichment of the reporter complexes out of excessive interferences, but also provided sufficient space for immobilized probes to avoid steric hinderance, thus improving the efficiency of the device. Together with the robust isothermal HCR strategy, this nucleic acid device realized an optimal detection limit of 0.14 fmol/L with simple testing steps completed within 3 h. Similarly, another group developed a biosensor based on a series of toehold-mediated strand displacement and DNA nanoclew-facilitated luminophore loading, in which the toehold referred to a short section of 5~8 nucleotides capable of initiating strand displacement [34] (Figure 3b). The detection procedure started with the hybridization of miR-21 and the exposed toehold 1 of the substrate strand, leading to the liberation of probe 1 and a further exposure of toehold 2. Assistant strands were then added to hybridize with toehold 2, thus triggering the displacement of both probe 2 and miR-21, releasing the target molecule into another cycle of reaction. As a result, the presence of miR-21 was successfully converted into a large amount of probe 2. These ssDNAs carried on the detection process by opening the stem-locked region of a hairpin structure sensor and consequently capturing DNA nanoassemblies which were loaded with sufficient signal tags benefiting from the condensed clew-like DNA structure. In brief, their biosensor design was mostly composed of nucleic acid nanomaterials, and realized an LOD of 0.65 fmol/L.

Mousavi et al. [35] designed a two-step detection method with two specific oligonucleotide probes based on the sequence of miR-16-5p, which was a novel and non-invasive urinary biomarker indicating AKI when its concentration increased. Probe I was a 23-nt sequence complementary to the 12–22 region of miR-16-5p and decorated with a C6SH group at 5’-end to be immobilized on Fe_3_O_4_ magnetic nanoparticles (MNPs). During Step One, Probe I could hybridize with miR-16-5p specifically while MNPs isolation helped to reduce the interference of other urinary components. On the other hand, probe II, which was a 23-nt nucleotide complementary to the 1–11 base region of miR-16-5p, was exploited in Step Two to hybridize with the complex isolated from Step One. Concretely, probe II was modified with a C3SH group at 3’-end to attach to the gold sensor surface. As a result, miR-16-5p could be indirectly but specifically captured by a pair of probes rather than a single one, and further detected by the sensory platform with minimum bias and high sensitivity. Further validation in clinical urine samples and success in distinguishing AKI samples from CKD ones demonstrated the efficiency of these label-free nucleic acid probes with a calculated LOD of 17 fmol/L. 

To date, a series of miRNAs have been proven to change significantly either in serum or urine of patients facing early AKI. For instance, urinary miR-494/miR-30c-5p/miR-200c increase significantly during the early phase of AKI while urinary miR-4640 is downregulated, all of which are based on investigations from clinical studies [64,65,66]. However, urinary miRNAs are usually low in concentration, and PCR, the conventional detection method, involves several limitations such as sophisticated sample preprocessing procedure and false-negative issues due to enzymatic problems [67]. Furthermore, some extant research have already explored the inspiring applications of novel nucleic acid nanotechnology such as framework nucleic acids in miRNA detection, but within other disease backgrounds such as pancreatic carcinoma and lung cancer, or limited to theoretical designs only, with very few similar attempts and proof in the field of AKI diagnostics [68,69,70,71,72]. Therefore, as the miRNA targets in those cancer or theoretical research had overlap with miRNA spectrum in AKI, combining more AKI-related miRNAs with either highly programmable probe-based sensors or state-of-the-art DNA nanoassemblies investigated in these literatures may pave a new way for earlier AKI recognition. In addition, some researchers have proposed the concept of miRNA ‘panel’ tests in AKI diagnosis, which may outperform a single miRNA and improve the detection accuracy [73]. Therefore, with the rapid development of nanotechnologies such as DNA molecular computation, a comprehensive nucleic acid-based biosensor integrating the detection of several AKI-related miRNAs at the same time can be prospected [74].

### 2.4. AKI Diagnostics via DNA Nanostructures

In addition to aptamers and probes that directly detect targets, DNA nanostructures, which referred to nanoscale structures made of DNA, also contribute to AKI diagnosis in an indirect way. DNA nanostructures can serve as scaffold for more complex and versatile functional structures, including DNA tiles and origami, and play an innovative role in biomedical applications [75]. Cai and co-workers realized to detect radiolabeled DNA tetrahedron nanostructure (DTN) in vivo, which was annealed from four rationally designed single strands, via dynamic positron emission tomography (PET) imaging [76]. Quick distribution in urinary tracts and efficient renal clearance of DTN could be seen in healthy mice, allowing one to evaluate renal function by using the kidney time-activity curve analyzed from PET. By further exploring DTN profiles in murine unilateral ureteral obstruction (UUO) model, the team found a distinct uptake and kinetic pattern of DTN between healthy and UUO kidneys: the perfusion from serum to renal cortex remained almost the same while the obstructed side showed a significant decrease in DTN movement from renal cortex to pelvis compared with normal kidneys. In other words, the disparity in excretion could implicate a stimuli-stressed kidney. This non-invasive detection method may not only provide dynamic monitoring for an underlying acute kidney injury, but also offer visual presentation as well as quantification of two split kidney function.

### 2.5. Nucleic Acid-Based Proteomic Investigation for AKI

Scouting for novel protein biomarkers for various disease spectrum has long been a pursuit in medical diagnostics. With the rapid development of nucleic acid technology, aptamers are gradually facilitating the discovery of biomarkers. SOMAScan and CELL-SELEX are two of the most explored methods so far. SOMAScan, named after its utilization of slow off-rate modified aptamers (SOMAmers) which contain unique motif and bind targets in their native conformation, is an unbiased proteomic profiling platform that outperforms traditional methods and can be applied in complex biological samples consisting of thousands of proteins [77,78]. 

Yu et al. [79] interrogated the proteomic patterns via SOMAScan in the serum samples of dialysis-dependent AKI (AKI-D) patients who survived or died in a certain period of days. They screened out 33 proteins elevated in the serum of AKI-D patients who died in the first 8 days compared with those who survived over 8 days, among which fibroblast growth factor-23 (FGF23), tissue plasminogen activator (tPA), and soluble urokinase plasminogen activator receptor were strongly associated with a higher mortality. In addition, during the 8th~28th day of AKI-D events, high plasma level of FGF23, tPA, and interleukin-6 were related to an increased mortality. This SOMAScan-based AKI research not only revealed several potential biomarkers that could discriminate AKI-D patients with danger, but also guided a way towards further understanding of the pathogenesis involved in AKI-D. 

Regardless of the strengths of the novel high throughput method for biomarker exploration, more validation in clinical trials is still needed in order to avoid false discoveries and facilitate its medical transformation. Liu et al. [80] laid emphasis in their TRIBE-AKI cohort-based research on evaluating the consistency between SOMAScan results and the traditional immunoassay results, and further verifying the findings with liquid chromatography-mass spectrometry measurements, similar to the previous insights provided by Parikh’s group [81]. What’s more, CELL-SELEX and SOMAScan are more commonly used in other renal diseases, such as end-stage kidney disease, CKD, and diabetic nephropathy etc. [82,83,84,85,86,87]. Though kidney-related, backgrounds of these diseases are still different with those of AKI. As in AKI, complex samples such as blood and urine are more commonly and easily accessible than specific diseased cell lines derived from biopsy. Thus, SOMAScan, together with sufficient verification, may play a more pivotal role in the field of AKI and further helps to develop unprecedented biomarkers in the near future.

## 3. Therapeutic Approaches of AKI Based on Nucleic Acid Nanotechnology

Pathophysiology of AKI is rather complex, including extensive tubular oxidative stress, excessive release of inflammatory cytokines, activation of complement system, ferroptosis, necroptosis, cell cycle arrest and so on [88,89,90,91]. Albeit the springing up of various preclinical research of AKI therapeutics, they are still impeded by several factors, such as the poor targeting ability and the subsequent low efficiency, causing off-target effects in other organs and adverse drug reaction due to a higher administration dose to meet the expected efficacy [92,93]. Furthermore, the immunogenicity and biostability have also been a concern. Benefiting from the excellent biocompatibility and a preferential renal clearance, nucleic acid nanotechnology has been increasingly interrogated in the field of AKI treatment. This section will introduce nucleic acid-based therapeutics targeting various underlying reasons in AKI pathogenesis in details, with a highlight on different nanovehicles realizing renal localization, including DNA nanostructures and several DNA-based synthesized biomaterials.

### 3.1. Therapeutics Targeting Oxidative Stress

**Figure 4:**
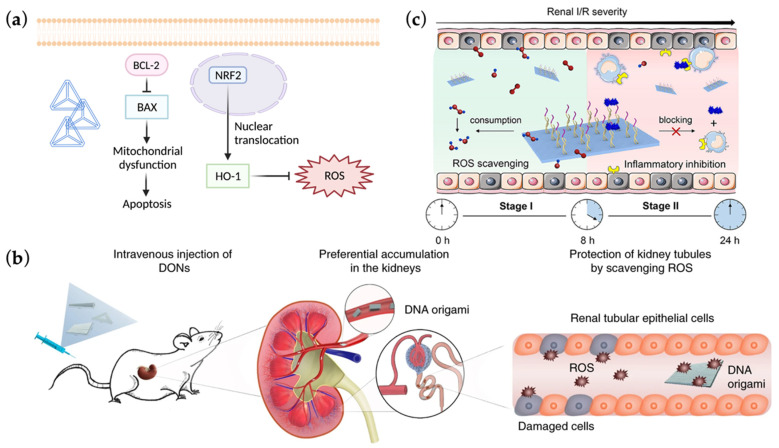
Mechanisms involved in AKI therapeutics targeting oxidative stress mediated by various nucleic acid nanotools. (**a**) Graphic display of how DNA tetrahedron nanostructure (DTN) functions as a potential AKI therapy through reducing oxidative stress and inhibiting apoptosis in renal tubular cells. Adapted with permission from [96] and created with BioRender.com (19 January 2022). Copyright © 2022, Elsevier; (**b**) Diagrammatic plot of how rectangular DNA origami nanostructures (Rec-DONs) accumulate in kidneys and neutralize reactive oxygen species (ROS) produced in AKI. Adapted with permission from [14]. Copyright © 2022, Springer Nature; (**c**) Schematic illustration of a sequential AKI therapy realized by nucleic acid nanodevices, in which Rec-DONs scavenge ROS during the first 8 h of ischemia-reperfusion (I/R) injury while the attached aptamers targeting complement component 5a are responsible for inflammatory inhibition after 8 h of I/R AKI. Adapted with permission from [103]. Copyright © 2022, American Chemical Society.

Rhabdomyolysis (RM), which is usually due to severe burns or traumatic injuries and characterized by damage of skeletal muscle and outflow of cell contents, can induce AKI (RM-AKI) [94]. The leakage of uric acid and iron-containing hemoprotein myoglobin can be pathogenic, resulting in oxidative stress and inflammasome activation in renal tubules, including the formation of reactive oxygen species (ROS) [90,95]. Lin’s team demonstrated an antioxidative role of DTNs (average size ≈ 11.7 nm) in RM-AKI [96] (Figure 4a). Researchers disclosed the ROS scavenging effect of DTN itself, which was consistent with previous studies showing DNA neutralization of oxidative substances [97]. What’s more, DTNs could transcriptionally upregulate nuclear factor E2 related factor 2/heme oxygenase-1 (NRF2/HO-1) axis, which was protective against oxidative stress, thus exerting further antioxidant effects [98]. On the other hand, DTNs also alleviated cell apoptosis through rescuing mitochondrial dysfunction and regulating mRNA level of apoptosis-related proteins B-cell lymphoma 2 (BCL2)/BCL-2-associated X protein (BAX). 

In 2018, Jiang et al. [14] explored the antioxidative potential of DNA origami nanostructures (DONs) towards RM-AKI in vivo through PET imaging (Figure 4b). Accordingly, DONs have risen as a successful implementation of DNA self-assembly, which are composed of an M13 phage-derived long ssDNA (scaffold), orchestrated by many short ssDNAs (staples), forming prescribed structures or patterns and realizing multiple functions, including addressable positioning of nanoparticles and targeted drug delivery [99,100,101]. In Jiang’s article, differently shaped DONs displayed preferential accumulation in kidney parenchyma over liver sequestration and were followed by whole-body clearance within 24 h, among which the rectangular DONs (Rec-DONs) seemed to accumulate the most (although not statistically significant). Rec-DONs (60 × 90 nm) could also pile up in renal tubules of experimental RM-AKI mice, yet with a longer excretion time, laying foundation for the retention-based treatment efficacy. A therapeutic dose (10 µg mouse^−1^) of Rec-DONs could obviously ameliorate AKI with a robust ROS-scavenging effect, displaying a similar effect to high-dose N-acetylcysteine, a clinically used antioxidant. With a proved biostability, non-toxicity and low immunogenicity, Rec-DONs might play a more significant role not only as an active drug but also as a useful carrier in AKI therapies upon further modifications and optimization. Of note, their work also hinted that biodistribution and biodynamics of nanostructures could be partially determined by their well-designed shapes and sizes, given the clue that more condensed origami structures, such as Rec-DONs, displayed a much better renal-over-liver accumulation than ssDNAs or partially folded scaffold strand, providing innovative perspectives for future design of DNA-based nanotherapeutics.

Ischemia-reperfusion (I/R) is one of the most common causes of AKI, in which the mismatch between oxygen supply and waste metabolism mediates renal mitochondrial dysfunction, leading to ROS production and a subsequent series of deleterious responses including inflammation and apoptosis [88,102]. In 2021, Chen et al. [103] also employed Rec-DONs to mitigate I/R AKI (Figure 4c). Based on their long retention time in kidneys (>12 h) as well as the timeline of pathophysiological progression in AKI, the team delicately introduced aptamers that could specifically block complement component 5a (C5a) to Rec-DONs, in which the ROS-sensitive DNA nanostructure itself contributed to antioxidative effects during the first 8 h while the lodged anti-C5a aptamers competitively bound C5a, thus suppressing further inflammatory responses 8 h after I/R AKI. Their nanodevice not only targeted double factors, but also realized a sequential and continuous treatment for AKI.

Keeping the above in view, these research disclosed a promising antioxidative effect of DNA nanostructures as well as its derivative devices.

### 3.2. Therapeutics Targeting Ferroptosis

**Figure 5:**
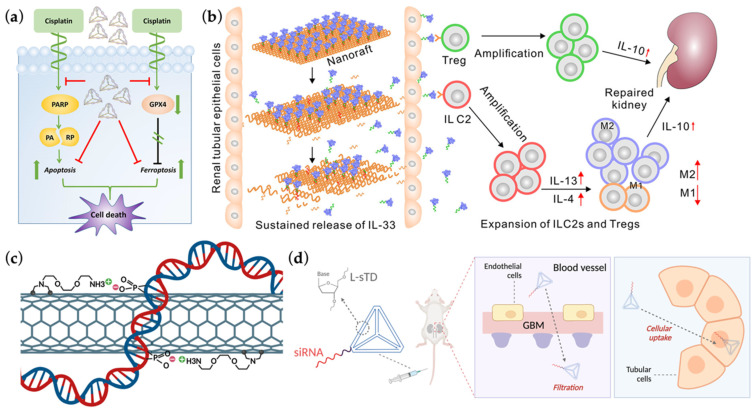
(**a**) Graphic display of the protective role DTNs play in cisplatin-induced AKI via inhibition of both ferroptosis and apoptosis. Adapted with permission from [108]. Copyright © 2022, American Chemical Society; (**b**) Sketch map of DON-based nanorafts rationally assembled with interleukin-33 and how the designed nanomachines function in immune-targeted AKI therapy. Adapted with permission from [110]. Copyright © 2022, American Chemical Society; (**c**) Schematic illustration of the conformation and noncovalent bonding between a fibrillar carbon nanotube and therapeutic short interference RNAs (siRNAs), realizing kidney-targeted delivery and AKI alleviation. Created with BioRender.com (19 January 2022); (**d**) Diagram of the incorporation of mirror DTNs and p53 siRNAs, in which DTNs contribute to renal distribution and tubular cell uptake while siRNAs are responsive for downregulating p53-related cellular apoptosis. Adapted with permission from [121] and created with Biorender.com (21 January 2022). Copyright © 2022, American Chemical Society.

Ferroptosis is a recently identified mode of regulated cell death dependent on accumulated reactive iron and lipid peroxidation [104,105]. It has also been reported to play a significant role in cisplatin-induced AKI (cis-AKI) and folic acid-induced AKI other than the commonly involved types of cellular death [106,107]. Wu’s group revealed DTNs (≈18.79 nm) possessed therapeutic potential against ferroptosis in cis-AKI, where it could rescue renal proximal tubular epithelial (HK-2) cells treated with ferroptosis-inducer in vitro through reducing lipid ROS as well as reversing the downregulation of glutathione peroxidase 4 (GPX4) in both transcription and translation level [108] (Figure 5a). Similar restoration of GPX4 expression was also observed in cisplatin-treated HK-2 cells, further accompanied by apoptosis inhibition via reducing the cleavage of poly (ADP-ribose) polymerase. Their work was the first who revealed the anti-ferroptosis effect of DTNs in AKI, paving a way for more nanotherapeutics targeting this pathway. Notice that, transcription of *GPX4* could be increased with NRF2 accordingly, and the study in Section 3.1 [96] happened to demonstrate an elevated mRNA level of NRF2 after DTN intervention in renal cells [96,109]. Whether the restoration of GPX4 expression in this research was due to DTN-mediated upregulation of NRF2 needs to be further investigated.

### 3.3. Therapeutics Targeting Immune Responses

Recently, Liu and Zheng’s team constructed a cytokine-based nanoraft targeting immune responses in I/R AKI [110] (Figure 5b). They programmed 42 capture strands rationally on the surface of Rec-DONs, followed by hybridization with DNA-modified interleukin-33 (IL-33), a renoprotective cytokine, thus realizing a precise and quantitative nanoassembly capable of releasing cytokine cargoes sustainably [111]. The team then validated their Rec-DON-functionalized nanomachine could accumulate in kidney for up to 48 h. Compared with free IL-33 therapy, their nanorafts managed to reach a better therapeutic effect in I/R AKI mice models with a lesser dosage and a lower administration frequency, in which the long-term kidney-localized release of IL-33 successfully expanded type 2 innate lymphoid cells and regulatory T cells, ameliorating the injured kidney. Of note, their invention could also accumulate in liver promptly after intravenous injection of nanorafts. Although no obvious hepatic side effects found in this study, further improvement was still expected to realize a more precise renal targeted delivery probably through some peptide modifications. Nevertheless, their precise design efficiently avoided the main limitations of conventional cytokine immunotherapy where free IL-33 had a rather short half-life and non-specificity to kidney-resident immune cells [112].

### 3.4. Therapeutics Targeting p53-Related Cellular Apoptosis

p53, a pivotal tumor-suppressor protein, has been extensively reported for regulating cellular apoptosis [113]. It is demonstrated in AKI that proximal tubular p53 can be pathogenic by inducing several vital genes related to cell death regulation [114]. On the other hand, short interference RNA (siRNA) is double-stranded RNA molecule composed of 21~25 nucleotides which can induce degradation of target RNAs in a sequence-specific manner, thus frequently functionalized in muting *p53* gene as a nanoscale nucleic acid tool [115]. Experiments have shown a beneficial effect of *p53*-targeted siRNAs towards ischemia and cisplatin-induced AKI, presenting a dose and time-dependent attenuation of apoptotic signaling [116]. However, the application of these *p53*-aiming siRNAs is still complicated with poor pharmacokinetics, susceptibility to nuclease degradation and off-target effects due to non-specific delivery, calling for more possible vehicle nanoplatforms and appropriate modifications aiming at more well-directed efficacy and fewer side effects in AKI treatment [92]. 

Recently, Tang and colleagues electrostatically loaded *p53* siRNA upon their previously explored chemical nanocarrier, polymeric C-X-C chemokine receptor 4 (CXCR4) antagonist (PCX), which was a linear polymer composed of phenylene-cyclam derivative and hexamethylene-bis-acrylamide and was about 127 nm in size [117,118]. Notice that, PCX not only blocked inflammation-related membranous CXCR4 from receiving upstream signals and transducing pathogenic responses, but also mediated endocytosis of the PCX/siRNA polyplexes upon membranous CXCR4-binding in injured tubular cells. With dual-functionality and durable renal retention in AKI, the nanoparticles favorably accumulated in injured kidney tubules and robustly ameliorated apoptotic events with a reduced *p53* siRNA dose of 0.6 mg/kg compared to naked ones. Alidori et al. [119] exploited ammonium-functionalized carbon nanotubes (fCNTs) (≈300 nm) in the dual-delivery of siRNAs aiming at *p53* and meprin-1β, another key factor involved in AKI procedure, to proximal tubular cells specifically [120] (Figure 5c). This nanocomplex was proved in murine cis-AKI models to prophylactically attenuate AKI with a cumulative siRNA dose of 0.4 mg/kg; meanwhile, its pharmacodynamic profile was evaluated in primates for the first time, which was consistent with the results in mice and might facilitate its clinical transformation. 

DNA nanostructure can also be employed as a cytosolic delivery tool for the anti-apoptotic oligonucleotides since the cargo siRNAs can easily satisfy the principle of base-pairing with the carrier. Thai et al. [121] prepared L-sTD, a mirror DNA tetrahedron with 10 bp on each side and sugar backbone modifications, demonstrated with a kidney-preferred distribution in vivo (Figure 5d). Meanwhile, the small size (≈6 nm) of L-sTD allowed itself to filter through glomerular base membrane freely and undergo megalin-mediated endocytosis by renal tubular cells. The team then applied siRNA targeting *p53* (siP53) to L-sTDs forming siP53@L-sTD via hybridization of two designed linker DNA strands and eventually proved its anti-apoptotic therapeutic effects for AKI in vivo through downregulating *p53* transcriptionally. Further experiments elucidated siP53@L-sTD outperformed naked siP53 mainly due to its successful escape from endosome entrapment, which was mediated by interaction between L-sTds and lipids at lowered pH within endosomes, resulting in membrane destabilization. This research managed to solve the main challenges of siRNAs by lowering the dosage (from 5 mg/kg to 0.25 mg/kg per injection) with a higher cellular uptake efficiency and minimizing off-target effects with kidney-specific delivery.

## 4. Conclusions and Outlook

Herein, nucleic acid nanotechnology harnessed for diagnostics and therapeutics in AKI is mainly discussed, in which the specific binding ability, diverse range of targets and easy incorporation into various biochemical platforms contribute to novel methods for AKI early recognition while the flexible designability, clear addressability, low immunogenicity, and potential renal retention play a pivotal role in disease intervention. The objective of this review is to summarize the existing discoveries so that more attention can be paid to this prospective field, considering the relatively small amount of concerned research to date. 

Of note, our review lays much emphasis on the functionalization of aptamers, a novel but robust kind of ‘chemical antibodies’ based on nucleic acids. Through rationally designed SELEX technology, certain aptamers can be screened out with high binding affinity and optimal specificity towards a variety of targets, such as proteins, peptides, metal ions, and even cells and viruses, which possesses advantages over conventional antibodies used at clinics. In addition, nucleic acids confer themselves to a series of more accessible and easy amplification strategies based on DNA hybridization or DNases, which can significantly lower the LOD, making the diagnostics faster and even earlier when detecting biomarkers of early AKI.

In spite of the robust performance mentioned above, nucleic acid nanotechnology-based diagnostic and therapeutic methods are still preclinical and may be complicated with multiple obstacles to eventual clinical transformation: (1) As the field combining AKI and nucleic acid nanotechnology is rather new, many of the experiments and models have been based on chemical buffer or artificial urine/serum, which warrants much more validation in authentic clinical AKI samples where these theoretical methods can really help; (2) The whole synthetic process of DNA nanostructures might be expensive due to highly demanding purification steps and non-ideal yield, which is a common challenge for many nucleic acid-based approaches. According to Jiang et al.’s research, a single dose of their Rec-DON-mediated AKI therapy required 80 dollars for the DNA materials only, so they proposed to improve their DNA assembly strategies in order to reduce the costs, such as DNA nanoassembly construction via Escherichia coli expression or mass production through bacteriophages [14]; (3) Although DNA biomaterials have been widely proved with decent biodegradability and compatibility in vivo, their biosafety should be once again considered when attached to inorganic platforms or modified with extra functional group. If possible, the fate of these foreign aids should be investigated other than only testing the viability on cellular level. On the other hand, strategies can also be expanded in terms of the renal disease: (1) As AKI is quite complex, when designing a therapeutic nanomachine, targeting double or even triple underlying pathogenic factors may present a much better efficacy than those with simple functionality; (2) As DNA-based smart drug delivery systems have been widely explored, nanodrugs encapsulated in DNA self-assemblies can be applied, which has barely been attempted in AKI to date. (3) In addition, when detecting AKI biomarkers by aptamers, more novel proteins can be selected as targets, such as kidney injury molecule-1 (KIM-1) which outperforms NGAL and cysC mainly adopted in this article in AKI early diagnosis. Moreover, as KIM-1 is a transmembrane protein rather specific to renal tubular cells, it may also help localize therapeutic nanoparticles to kidneys for more precise treatment; (4) Meanwhile, as the global standards emphasize the significance of stratifying different AKI stages so as to provide more suitable and appropriate therapies, whether the novel diagnostics mentioned above can help grade patients warrants further exploration. In fact, in Xu et al.’s research, they managed to detect urinary miR-21 in AKI patients with their nucleic acid probe-functionalized ECL biosensor and realized to prove urinary miRNA-21 gradually increased along with the progression of AKI from stage 1 to 3 [33]; (5) Last but not least, the extant traditional AKI diagnostics should still be paid much attention to as they are the basis of multifarious official instructions, such as the authentic Kidney Disease: Improving Global Outcomes guidelines.

Nevertheless, limitations allow further modifications. We believe upon future optimization, challenges can be overcome, and inspiring findings can be prospected in the convergence of nucleic acid nanotechnology and AKI, or even other renal disease spectrum.

## Figures and Tables

**Table 1:** A summary of extant nucleic acid nanotechnology-based diagnostics towards AKI-related biomarkers.

Diagnostic Targets		Type of Receptor	Type of Surface or Electrodes	Methods	Samples	LOD	Range of Detection	Refs
Proteins	NGAL	NGAL antibody and DNA aptamer	/	ELAA	Buffer and Human AKI urine	30.45 ng mL^−1^	125~4000 ng mL^−1^	[19]
		FAM-labelled DNA aptamer	PDANS	Fluorescence detection and DNase I-aided amplification	HK-2 cells and Mice AKI urine	6.25 pg mL^−1^	12.5~400 pg mL^−1^	[20]
		DNA aptamer	GNP-modified biochip	SWV	Buffer	0.07 pg mL^−1^	0.1~10 pg mL^−1^	[21]
		Redox reporter-modified DNA aptamer	Gold electrodes	SWV	Artificial and Human urine	2 and 3.5 nM	Covers 2~32 nM	[22]
		DNA aptamer	AgNP IDE	EIS	Buffer and Artificial urine	10 and 3 nM	3~30 and 3~300 nM	[23]
		RNA aptamer	Microcantilever sensor	Differential interferometry	Buffer	96 ng mL^−1^	Covers 100~3000 ng mL^−1^	[24]
	CysC	DNA-linked antibody pair	AuNP-functionalized Fe_3_O_4_ and G/mRub	ECL measurement and DNA strand displacement-mediated amplification	Buffer and Human serum	0.38 fg mL^−1^	1.0 fg mL^−1^~10 ng mL^−1^	[25]
		FAM-labelled DNA aptamer	GO	Fluorescence detection and DNase I-aided amplification	Buffer and Mice AKI urine	0.16 ng mL^−1^	0.625~20 ng mL^−1^	[26]
		CysC antibody and DNA aptamer	/	Competitive ELASA	Buffer and Human serum	216.077 pg mL^−1^	/	[6]
		CysC antibody and DNA aptamer	/	Quantitative fluorescence LFA	Buffer and Human urine	0.023 μg mL^−1^	0.023~32 μg mL^−1^	[27]
	RBP4	DNA aptamer	Gold chip	SPR	Artificial serum	1.58 µg mL^−1^	/	[28]
	Albumin	Cy5-labelled DNA aptamer	GO	Fluorescence detection	Human urine and human serum	0.05 µg mL^−1^	0.1~14.0 µg mL^−1^	[29]
		DNA aptamer	Magnetic beads	DPV aided with methylene blue solution	Artificial and Human urine	0.93~1.16 µg mL^−1^ (pH-related)	10~400 µg mL^−1^	[30]
Small molecules	Urea	DNA aptamer	AuNP	Colorimetric detection	Milk sample	20 mM	20~150 mM	[31]
		DNA aptamer	CNTs/NH_2_-GO	DPV	Buffer and Human urine	370 pM	1.0~30.0 nM and 100~2000 nM	[32]
Nucleic acids	miR-21	DNA probes	Magnetic beads	ECL measurement and HCR-mediated amplification	Buffer and Human AKI urine	0.14 fM	1 fM~1 nM	[33]
		DNA probes	/	ECL measurement TMSD- and DNA NCs-aided amplification	Buffer and Cell lines	0.65 fM	1 fM~100 pM	[34]
	miR-16-5p	DNA probes	Capped gold nanoslit	SPR	Human AKI urine	17 fM	Up to nanomolar	[35]

Cys C, cystatin C; RBP4, retinol binding protein 4; LOD, limit of detection; ELAA, enzyme-linked aptamer analysis; FAM, 5-carboxyfluorescein; PDANS, polydopamine nanosphere; HK-2, human kidney 2 cells; GNP, graphene nanoplatelets; SWV, square wave voltammetry; AgNP, silver nanoparticle; IDE, interdigitated electrode; EIS, electrochemical impedance spectroscopy; AuNP, gold nanoparticle; G/mRub, monolayer rubrene functionalized graphene composite; ECL, electrochemiluminescence; GO, graphene oxide; ELASA, enzyme-linked aptamer sorbent assay; LFA, lateral flow assay; SPR, surface plasmon resonance; Cy5, cyanine 5; DPV, differential pulse voltammetry; CNT, carbon nanotubes; NH2-GO, amine-functionalized graphene oxide; HCR, hybridization chain reaction; TMSD, toehold-mediated strand displacement; DNA NCs, DNA nanoclews.

## Data Availability

Not applicable.
